# Sun Exposure and Protection Habits in Pediatric Patients with a History of Malignancy

**DOI:** 10.1371/journal.pone.0137453

**Published:** 2015-09-08

**Authors:** Yael Levy-Shraga, Rinat Cohen, Michal Ben Ami, Yonatan Yeshayahu, Vered Temam, Dalit Modan-Moses

**Affiliations:** 1 Pediatric Endocrinology Unit, The Edmond and Lily Safra Children's Hospital, Chaim Sheba Medical Center, Tel-Hashomer, Ramat-Gan, 52621, Israel; 2 Primary Care Division, Meuhedet Health Services, Tel-Aviv, Israel; 3 The Sackler School of Medicine, Tel-Aviv University, Tel-Aviv, 69978, Israel; 4 Department of Pediatric Hematology-Oncology and BMT, The Edmond and Lily Safra Children's Hospital, Tel-Hashomer, Ramat-Gan, 52621, Israel; A*STAR, SINGAPORE

## Abstract

**Background:**

Survivors of childhood cancer are at high risk for developing non-melanoma skin cancer and therefore are firmly advised to avoid or minimize sun exposure and adopt skin protection measures. We aimed to compare sun exposure and protection habits in a cohort of pediatric patients with a history of malignancy to those of healthy controls.

**Methods:**

Case-control study of 143 pediatric patients with a history of malignancy (aged 11.2±4.6y, Male = 68, mean interval from diagnosis 4.4±3.8y) and 150 healthy controls (aged 10.4±4.8y, Male = 67). Sun exposure and protection habits were assessed using validated questionnaires.

**Results:**

Patients and controls reported similar sun exposure time during weekdays (94±82minutes/day vs. 81±65minutes/day; p = 0.83), while during weekends patients spent significantly less time outside compared to controls (103±85minutes/day vs. 124±87minutes/day; p = 0.02). Time elapsed from diagnosis positively correlated with time spent outside both during weekdays (r = 0.194, p = 0.02) and weekends (r = 0.217, p = 0.01), and there was a step-up in sun exposure starting three years after diagnosis. There was no significant difference regarding composite sun protection score between patients and controls. Age was positively correlated with number of sunburns per year and sun exposure for the purpose of tanning, and was negatively correlated with the use of sun protection measures.

**Conclusions:**

Although childhood cancer survivors are firmly instructed to adopt sun protection habits, the adherence to these instructions is incomplete, and more attention should be paid to improve these habits throughout their lives. Since sunlight avoidance may results in vitamin D deficiency, dietary supplementation will likely be needed.

## Introduction

Sun exposure is the major environmental risk factor for skin cancers, both melanoma and non-melanoma [1]. Therefore, the World Health Organization [2], the American Academy of Dermatology [3] and the American Academy of Pediatrics [4] have issued guidelines advocating sun-protection measures in addition to avoiding tanning and sunbathing [5].

Survivors of childhood and adolescent cancer, especially those who were treated with radiotherapy, are at a relatively high risk for developing non-melanoma skin cancer [6] and therefore are firmly advised to avoid or minimize sun exposure and adopt skin protection measures such as applying sunscreen and wearing protective clothing, hats and sunglasses. Still, little is known about the adherence to these recommendations [7–12].

In the current study we describe sun exposure and protection habits of pediatric patients with a history of malignancy compared to those of healthy children, and identify factors associated with sun behavior. We hypothesized that sun exposure would be decreased, and that usage of sun–protection measures would be increased in patients compared to healthy controls. Since about 12% of our patients were Jewish ultra-orthodox, whose lifestyle encourages a conservative dress code with indoor scholarly activity and minimal skin exposure, we compared sun-habits of ultra-orthodox and non-religious participants of both study groups.

## Methods

### Participants

The study was carried out in the pediatric hematology-oncology outpatient clinic at a large tertiary children's hospital.

Oncology patients or their parents (for children younger than 11 years of age) were asked during a routine visit to fill out a questionnaire regarding sun habits. All patients 2–21 years old, during active anti-cancer treatment or after, were offered participation in the study. Demographic data including age, gender, time of diagnosis, disease type, and anti-cancer treatment were obtained from the patients' charts [13].

The control group comprised children and adolescents aged 2–21 years attending six primary care pediatric clinics. Children with chronic diseases were excluded from the control group. The control group questionnaires were anonymous, however parents were asked to specify their child's age, gender and degree of religious observance.

The study was approved by the institutional review committee, and written informed consent was obtained from all participants or their parents (in the case of minors).

### Sun habits

Sun exposure and sun protection habits were evaluated using a validated questionnaire referring to current habits [14, 15]. Forward/backward translations of the original questionnaire were completed by expert translators. Sun exposure habits were measured by asking the respondents to indicate the average number of hours they spent outside in the summer between 10 a.m. and 4 p.m. both for weekdays and weekends. Participants were also asked how many times they had red or painful sunburns during the previous year. Sun protection habits were assessed by measuring 5 protective behaviors (using sunscreen, wearing a shirt with sleeves, wearing sunglasses, staying in the shade and wearing a hat) on a 5-point ordinal scale (1 = never to 5 = always). A composite sun protection habits score was calculated by averaging responses to the 5 items [15]. In addition we asked the respondents how often they sunbathed for the purpose of tanning.

### Data analysis

The initial analysis included estimations of mean, standard deviations (SD), and frequency distribution. Comparisons between patients' and control groups and between ultra-orthodox and secular participants were made using the unpaired t-test. To assess the correlation between age or years from diagnosis and sun exposure and protection habits we used the Pearson correlation test. Results were considered significant if the two-sided p-value was <0.05. Calculations were performed using SPSS 15.0, a statistical software package.

## Results

### Patients' characteristics

Patients' characteristics are presented in Table 1. Two hundred and ninety three children and adolescents aged 2–21 years participated in the study: 143 participants with a history of malignant disease (mean interval from diagnosis 4.4±3.8 years; 3.5±3.2 years from the end of active treatment), and 150 healthy controls. Within the patients’ group, 32 participants were still actively treated at the time of the study.

**Table 1:** Characteristics of patients with a history of malignancy compared to healthy control.

	Patients (n = 143)	Controls (n = 150)	p-value
Age	11.2±4.6	10.4±4.8	NS
Male (%)	68 (46.9)	67 (45.9)	NS
Ultra-orthdox (%)	17 (11.7)	21 (14.4)	NS
Diagnosis			
Leukemia	57 (39.9%)		
Lymphoma	16 (11.2%)		
Brain tumor	31 (21.6%)		
Solid tumor	27 (18.9%)		
Other	12 (8.4%)		

### Sun exposure habits

**Fig 1:**
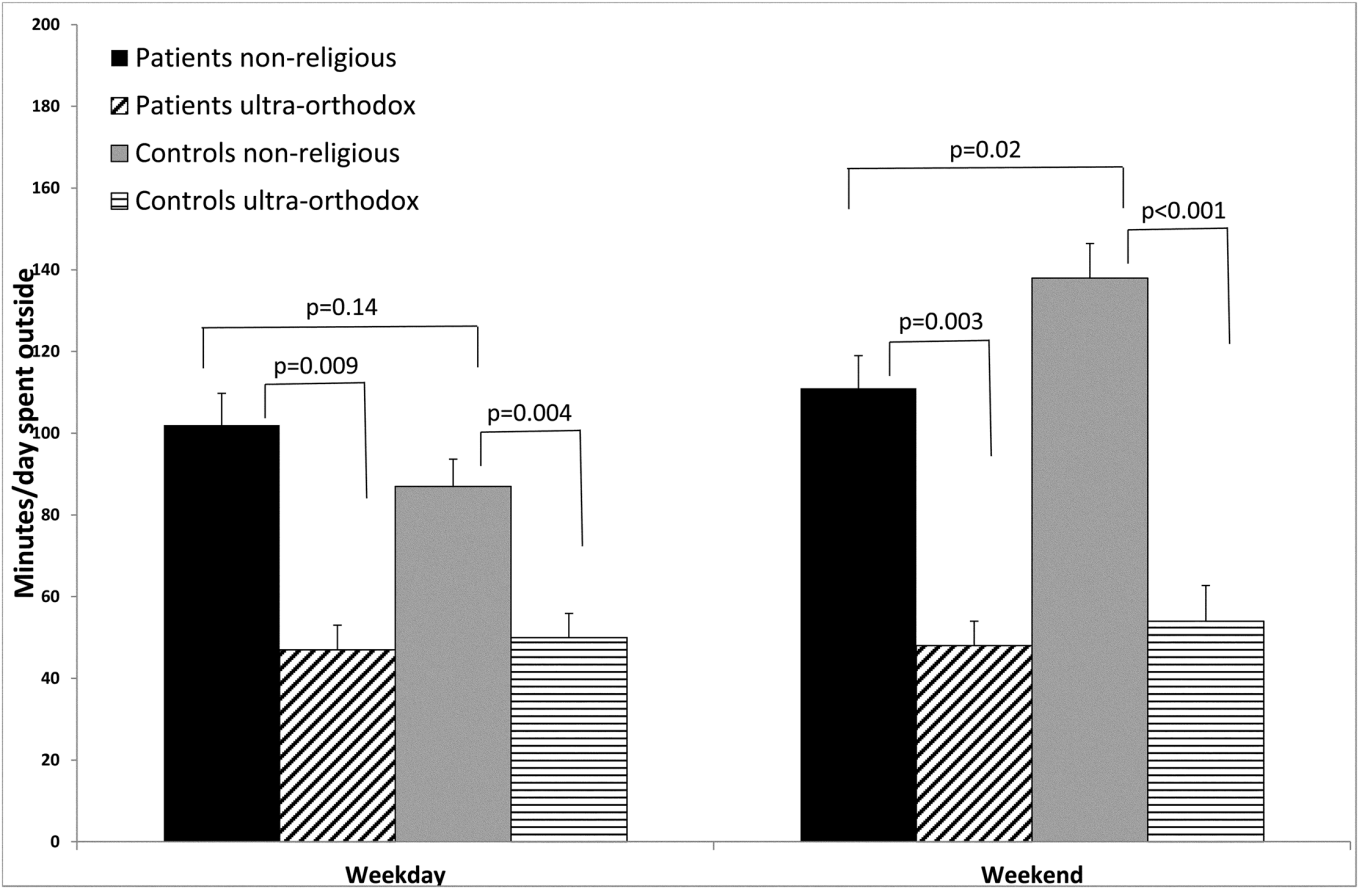
Sun exposure of patients compared to controls (Mean+SEM).

Patients and healthy controls reported a similar time of sun exposure during weekdays (94±82 minutes/day vs. 81±65minutes/day; p = 0.83), while during the weekend patients spent significantly less time outside compared to controls (103±85 minutes/day vs 124±87minutes/day; p = 0.02) (Table 2) (Fig 1). Ultra-orthodox participants reported significantly less time spent outside both during weekdays as well as during the weekends, whether they belonged to the patients group or to the control group (p<0.01 for all comparisons) (Fig 1).

**Table 2:** Minutes/day (mean±SD) spent outside in the summer between 10 a.m and 4 p.m of the two study groups.

	Patients (n = 143)	Control (n = 150)	p-value
**Weekday**			
**Total**	**94±82**	**81±65**	**0.83**
**Non-religious**	**102±86**	**87±71**	**0.14**
**Ultraorthodox**	**47±24**	**50±27**	**0.7**
**Weekend**			
**Total**	**103±85**	**124±87**	**0.015**
**Non-religious**	**111±88**	**138±90**	**0.02**
**Ultraorthodox**	**48±24**	**54±40**	**0.6**

Patients were significantly less likely than controls to get sunburned (0.2±0.7times/year vs. 0.4±0.9times/year; p = 0.01). There was no difference in the reported frequency of sunbathing for the purpose of tanning between the two groups (mean 1.4±0.8 in both groups; score 1 = never to 5 = always).

### Sun protection habits

**Fig 2:**
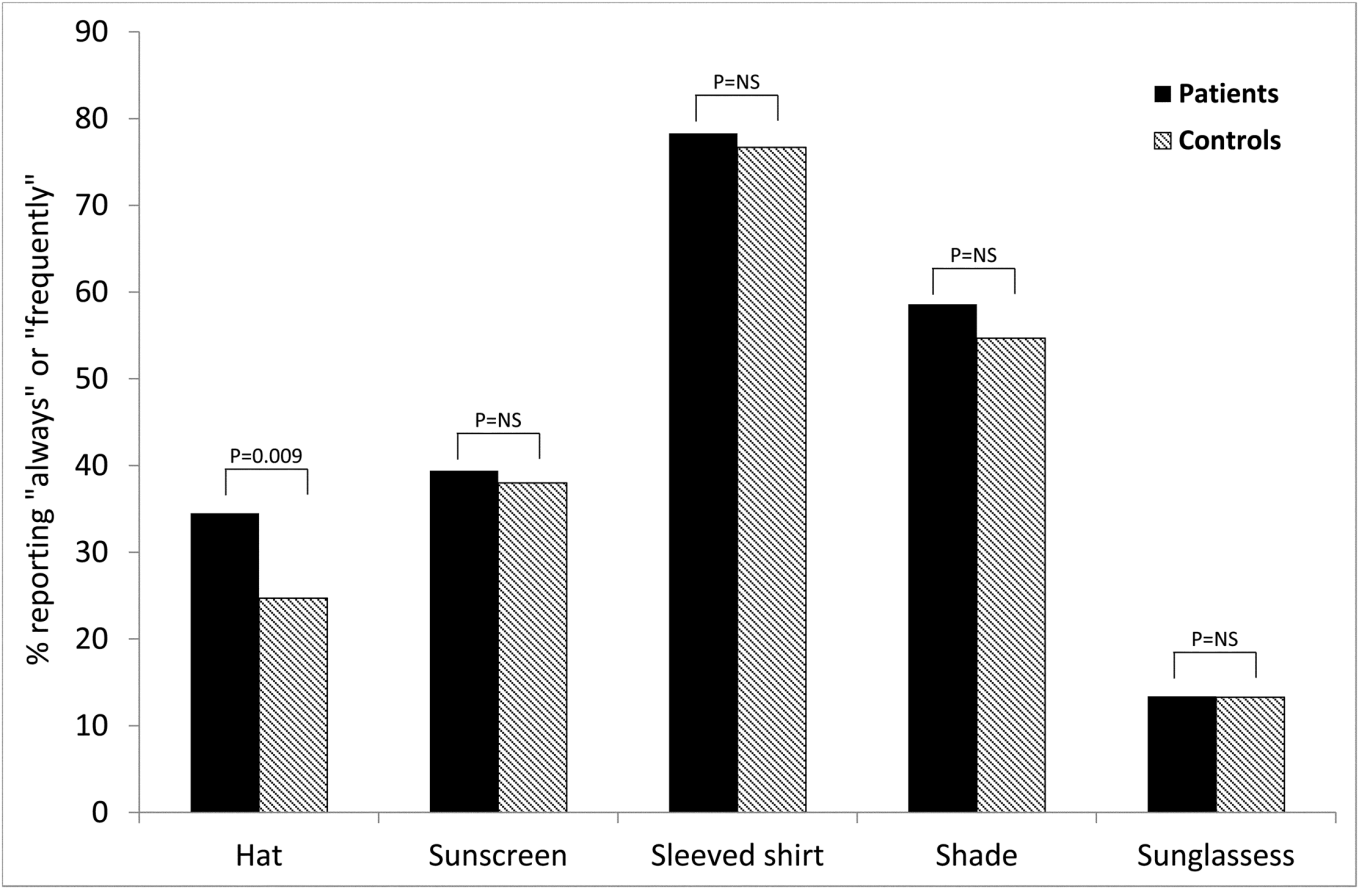
Sun protection habits of patients compared to controls.

Patients were more likely than controls to wear a hat when in the sun (34.5% vs. 20.7% reporting "always" or "frequently"; p = 0.009). There was no difference between the two groups regarding the frequency of using sunscreen (39.4% vs. 38% reporting "always" or "frequently"), wearing a shirt covering the shoulders (78.3% vs. 76.7%), staying in the shade (58.6% vs. 54.7%) or wearing sunglasses (13.4% vs. 13.3%) (Fig 2). There was no difference in the composite sun protection score (mean 3.2±0.6 vs. 3.0±0.5; p = 0.07; score 1 = never to 5 = always). There was no correlation between the composite sun protection habits score and duration of sun exposure, neither during weekdays(r = 0.044, p = 0.43) nor during the weekend (r = 0.037, p = 0.50).

Within the patients group, ultra-orthodox participants had a significantly lower sun protection score compared to non-religious participants (2.8±0.3 vs 3.2±0.6, p = 0.003).

Similar results were observed when ultraorthodox participants were compared to non-religious participants within the control group (2.8±0.3 vs 3.1±0.5, p = 0.015).

### Effect of active treatment and time elapsed from diagnosis

**Fig 3:**
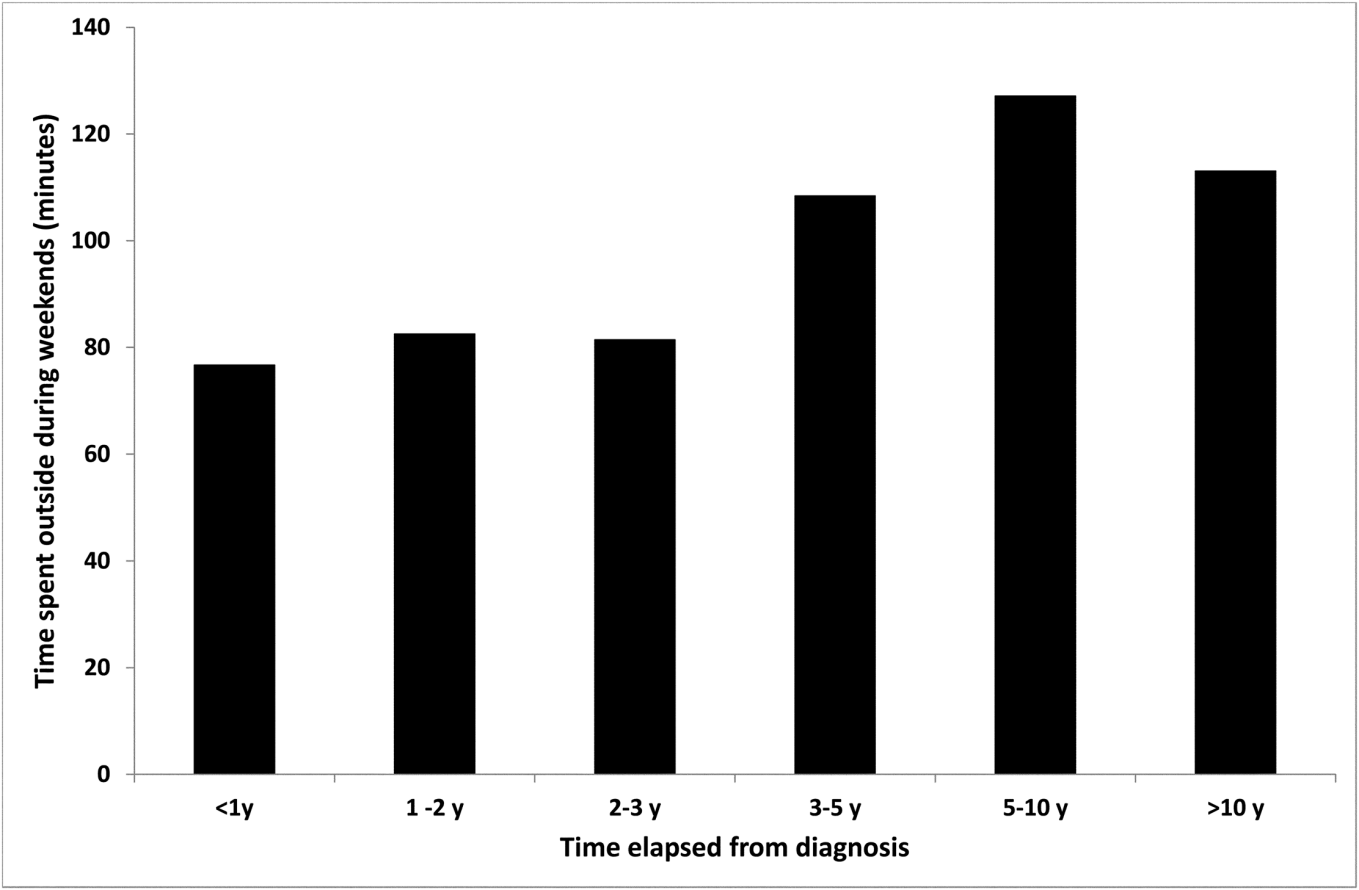
Time spent outside during the weekend according to time elapsed from diagnosis (patients' group only).

Patients who were still under active treatment spent significantly less time outside during the weekend compared to patients who completed therapy (83±83minutes vs. 108±85 minutes, p = 0.04). Time elapsed from diagnosis was positively correlated with time spent outside each day both during weekdays (r = 0.194, p = 0.02) and during the weekend (r = 0.217, p = 0.009), as well as with sun exposure for the purpose of tanning. The main increase in time spent outside occurred after three years from the time of diagnosis (Fig 3).

**Fig 4:**
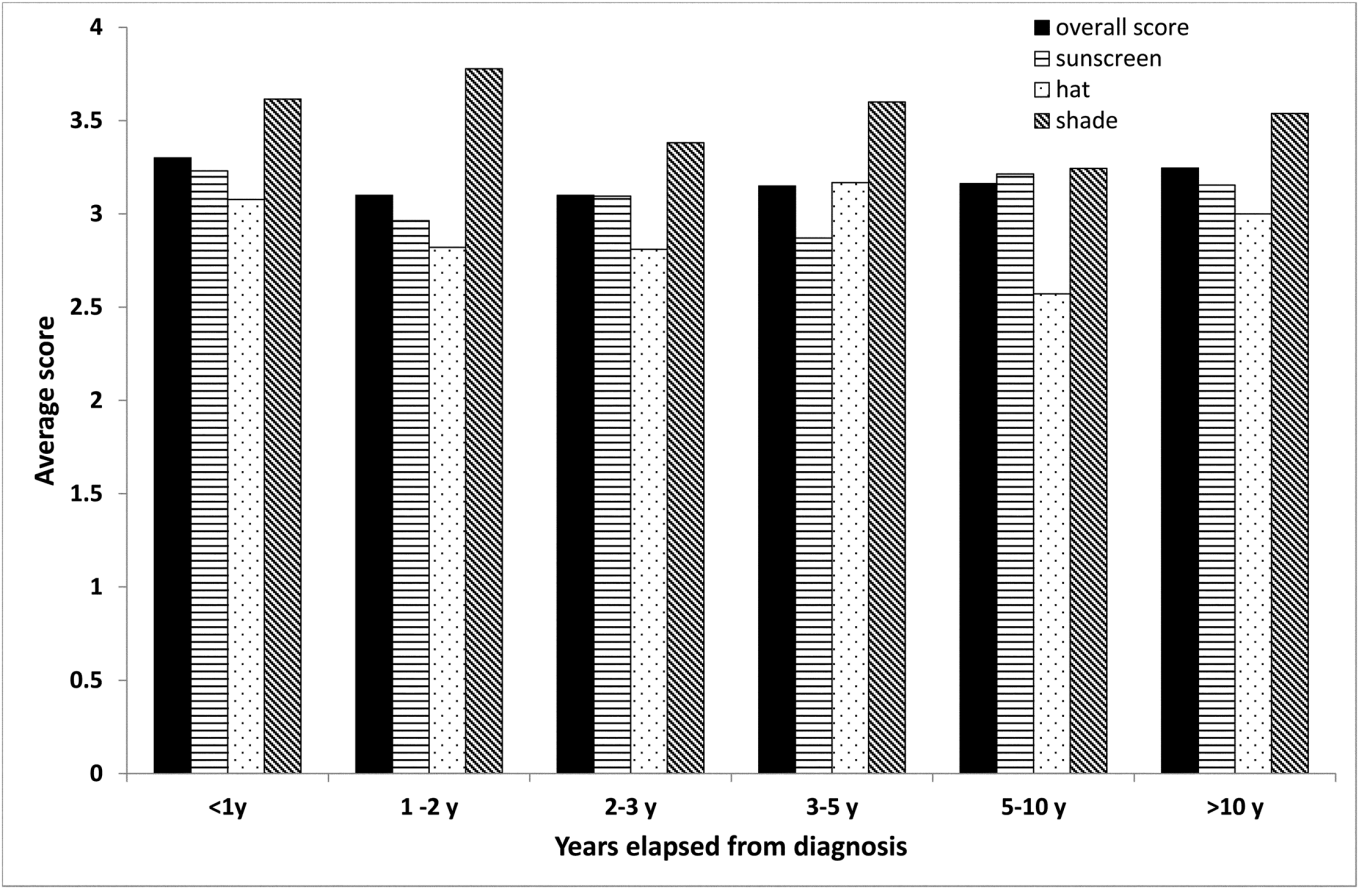
Use of sun protection measures according to time elapsed from diagnosis (patients' group only).

Time elapsed from end of treatment was negatively correlated (r = -0.21, p = 0.023) with staying in the shade but not with any of the other sun-protection habits (Fig 4).

### Correlation between age and sun habits

No correlation was found between age and time spent outside each day. However, age was positively correlated with number of sunburns per year (r = 0.15, p = 0.009), and sun exposure for the purpose of tanning (r = 0.424, p<0.001) Age was negatively correlated with use of sunscreen (r = -1.38, p = 0.018), wearing a hat (r = -0.327, p<0.001), staying in the shade (r = -0.126, p = 0.032) and the composite protection score (r = 0.147, p = 0.012).

## Discussion

In the current study we compared sun exposure and protection habits in a cohort of pediatric patients with a history of malignancy to those of healthy controls, and identified factors associated with sun behavior in this population. Patients and healthy controls reported a similar duration of sun exposure during weekdays, while during the weekend patients spent significantly less time outside compared to controls. A possible explanation for this finding is that sun-exposure during week-days is dictated mainly by the school-day schedule, whereas outdoor activities during the weekend are actively chosen by the child and the family and are thus more likely to be influenced by disease-associated factors such as energy level or a wish to avoid sun exposure. Time elapsed from diagnosis was positively correlated with sun exposure both during weekdays and weekends. Patients were more likely than controls to wear a hat when in the sun but there were no differences between the two groups regarding other sun protection measures. Using the composite sun protection score, there was only a trend toward a difference in favor of the study group (3.2±0.6 vs. 3.0±0.5; p = 0.07). This latter finding indicates that although patients are instructed to adopt sun protection habits, the adherence to these instructions is incomplete and should be improved.

Survivors of childhood and adolescent cancer are at high risk for developing non-melanoma skin cancer. In a report from the Childhood Cancer Survivor Study (CCSS), non-melanoma skin cancer accounted for 41% of all confirmed subsequent cancers in the cohort and melanoma for 3% [7]. There was a linear dose-response relationship between radiation therapy and risk of basal cell carcinoma, with an odds ratio of 39.8 for subjects who received 35 Gy or more to the skin site [6, 16]. These alarming rates indicate that childhood cancer survivors with a history of radiation treatment are especially susceptible to skin cancer and may subsequently increase their risk through sun exposure.

We identified only three previous studies in adults and three pediatric studies assessing sun behaviors in survivors of childhood cancer. In a report from the Swiss CCSS fewer adult survivors than controls reported protecting themselves from sun exposure (78% vs 87%)[8]. In contrast, adult survivors from the USA CCSS showed similar patterns of sunscreen use compared to their siblings (67% vs 66%), and survivors were significantly less likely to report having sunbathed or used artificial tanning in the previous year [7]. Finally, in a British study assessing sun behavior as a component of a Health Behavior Index, young adult survivors of childhood cancer led a healthier lifestyle compared to controls. However, sun behavior was assessed using a single question regarding number of sunburns in the year preceding the study [9].

As for sun habits in children and adolescents with a history of malignancy, previous smaller studies suggested low compliance with sun behavior recommendations. In a study comprising 75 adolescent survivors of childhood cancer, 37% were non-adherent to sun protection recommendations [10]. In a study comprising children and adults who had undergone hematopoietic stem cell transplantation (HSCT), the majority of participants reported “rarely” or “never” using sunscreen, and only 16% of the 44 children and adolescents reported “always” using sunscreen. There were no significant differences in average daily sun exposure by sunscreen use category [12]. Both studies did not include a control group. Finally, a study comprising 22 pediatric patients undergoing allogeneic HSCT, found that only 14% of the patients reported "always" using sunscreen, and 50% of the patients reported "never" using sunscreen. Consistent with our own findings, time spent outside and skin exposure scores were significantly lower for patients compared to controls [11]. Compared with these studies, participants in our study reported a higher rate of compliance with sun recommendations, with 39.4% of patients and 38% of controls reporting "always" or "frequently" using sunscreen. This finding may reflect a heightened awareness of the need for sun protection among the general population due to the fact that Israel is exposed to a relatively high level of UV-B radiation [17].

We identified several factors associated with sun behavior. Time elapsed from diagnosis was positively correlated with time spent outside each day both during weekdays and during the weekend, as well as with sun exposure for the purpose of tanning. This correlation might be explained by the fact that patients closer in time to the diagnosis are sicker and thus less active. Another possible explanation is that the adherence to the recommendations decreases with time, as in our center instruction regarding sun avoidance/protection was given at the time of diagnosis by information leaflets as well as by the treating hemato-oncologist and the admitting nurse, but there was no formal reinforcement of sun-advice after completion of treatment. There seemed to be a step-up in sun exposure starting three years after diagnosis, possibly coinciding with recovery and return to full activity. This might be an important time-point for educational intervention and reinforcement of sun education. Age correlated negatively with the composite sun protection score, and correlated positively with sunbathing for the purpose of tanning. Accordingly, age correlated positively with the number of sunburns per year. This is consistent with other reports suggesting that adolescents comprise a special risk group with a positive attitude toward tanning [18–20]. Finally, ultra-orthodox participants, although reporting spending less than half the time outside than non-religious participants, had a significantly lower sun protection score suggesting a need for "sun smart" education in this population.

Sun avoidance and sun protection practices may lead to inadequate vitamin D levels, as the body's requirements of vitamin D are primarily obtained by skin exposure to UVR [21, 22]. We have previously reported vitamin D deficiency and insufficiency (<20 ng/ml) in about 50% of this patient cohort, with a positive correlation between the amount of sun exposure and vitamin D levels [13]. Similar findings have been recently observed by other investigators [11, 23–25]. These findings are consistent with the decreased sun exposure time observed in the patients' group in the current study as well as in others. Decreased vitamin D levels have been associated with increased risk for many chronic diseases [26] as well as with non-skin cancer incidence or survival [27]. Furthermore, clinical trials demonstrated reduced cancer incidence [28] and improved survival [29] with vitamin D supplementation, and laboratory studies demonstrated anti-cancerous effects of 1;25(OH)_2_D_3_, the physiologically active form of vitamin D [30–33]. In light of these findings, some investigators suggested modifying guidelines to allow “sensible” sun exposure enabling adequate synthesis of vitamin D [26, 34]. However, since liberalizing the sun safe message may offer people an excuse to over-expose, current guidelines discourage intentional sun exposure to induce vitamin D production, especially in high risk groups [3, 4] such as childhood cancer survivors.

Dietary vitamin D may be beneficial to overcome lack of sunlight exposure. However, dietary sources of vitamin D are scarce [21], and vitamin D intake depends on food fortification and supplements [35, 36]. In this respect, in our previous study only 8% of the pediatric patients with a history of malignancy reported taking vitamin D supplements [13]. Similar results were observed by other investigators in children and adolescents undergoing HSCT [11]. Special attention should be given to individuals that avoid sun exposure due to cultural reasons and clothing, such as the ultraorthodox in our population [37].

Some limitations of our study should be acknowledged. As with any study based on questionnaires, recollection bias could skew the results. Secondly, the age range is broad and obviously sun habits differ with age. For this reason we have also analyzed the results according to age.

In conclusion, although our patients were instructed to adopt sun protection habits the adherence to these instructions is incomplete. Since similar results were observed in other cohorts of childhood cancer survivors [7, 8, 10–12], we suggest that more attention should be paid to improve these habits throughout their lives. Patients who are more than three years after diagnosis and adolescents may be target groups for "sun smart" educational intervention. Since we consider our patients to be particularly vulnerable to the adverse effects of vitamin D deficiency in relation to bone health as well as possible extraskeletal effects in the context of malignancy, they require close monitoring and supplementation in order to optimize their vitamin D levels.

## List of Abbreviations

CCSS: Childhood Cancer Survivor Study
HSCT: hematopoietic stem cell transplantation
UVR: ultraviolet radiation
